# Altered Sensorimotor Cortical Activities During Forearm Movement in Humans With Spinal Cord Injury

**DOI:** 10.1111/cns.70601

**Published:** 2025-09-07

**Authors:** Bo Li, Honghao Liu, Guanghui Li, Yiran Lang, Rongyu Tang, Fengbao Yang

**Affiliations:** ^1^ School of Information and Communication Engineering North University of China Taiyuan China; ^2^ School of Mechatronical Engineering Beijing Institute of Technology Beijing China; ^3^ Department of Neuroscience, Faculty of Health and Medical Sciences University of Copenhagen Copenhagen Denmark; ^4^ Beijing Institute of Technology School of Medical Technology Beijing China; ^5^ Institute of Semiconductors Chinese Academy of Sciences Beijing China

## Abstract

**Aims:**

Decoding the motor intention by electroencephalography to control external devices is an effective method of helping spinal cord injury (SCI) patients to regain motor function. Still, SCI patients have much lower accuracy in the decoding of motor intentions compared to healthy individuals, which severely hampers the clinical application. However, the underlying neural mechanisms are still unknown. The aim of this paper is to investigate changes in cortical activities after SCI, which are direct determinants of motor decoding performance.

**Methods:**

We performed source localization analyses to investigate the changes in motor‐related cortical regions after SCI. Moreover, we measured the power spectral density values and event‐related spectral perturbation values to assess the characteristics of the changes in cortical potentials.

**Results:**

We found that motor‐related independent component clusters decreased while attempting upper limb movement after SCI. Additionally, the activities in the sensorimotor cortex obviously attenuated after SCI. theta, alpha, and beta band power modulation obviously altered in SCI individuals. Furthermore, the latency of the largest power increase or decrease between SCI and healthy individuals demonstrated obvious differences.

**Conclusion:**

Our findings revealed changes in motor‐related cortical areas after SCI, which were crucial for improving the accuracy of motor intention decoding and advancing the clinical application.

## Graphical abstract

The research found that motor‐related independent component clusters were significantly decreased while attempting upper limb movement after SCI, including the decrease in activated areas of primary motor cortex and the absence of supplementary motor cortex. Additionally, the activities in the sensorimotor cortex were significantly attenuated after SCI.

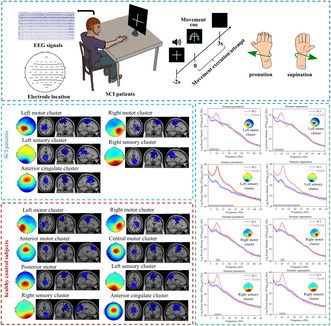

## 1 Introduction

Spinal cord injury (SCI) usually leads to severe motor dysfunction. However, there is currently no effective way to repair the injured spinal cord. Decoding the motor intention by electroencephalography (EEG) to control external devices has become the primary means of assisting movement. However, current studies on the decoding of movement have shown that SCI patients have a much lower level of accuracy in the decoding of motor intentions compared with healthy people [1, 2, 3], which severely hampers the clinical application. This is likely due to the reorganization of the functional areas of the cerebral cortex after SCI [4], which leads to changes in their functional activities.

In humans with SCI, existing and new areas within the motor, somatosensory, and parietal cortex, as well as the thalamus, basal ganglia, and cerebellum, show increased activity during motor execution compared to healthy controls [5, 6, 7]. These increased activities were characterized by a marked shift towards de‐efferented motor areas, secondary motor areas, and the somatosensory cortex [8, 9, 10]. The activation of new secondary motor areas immediately after injury may reflect their involvement in the development of new motor strategies. In addition, electrophysiological studies in people with SCI have shown that transcranial magnetic stimulation of the primary motor cortex results in larger motor cortical maps in muscles where motor neurons are located close to the site of injury compared to uninjured controls [11]. Resting‐state brain network studies have shown that cortical activation patterns in sensorimotor networks are dynamically reorganized after SCI, with reduced functional connectivity in motor and sensorimotor cortical areas in SCI patients compared to controls [12], and increased connectivity in the sensorimotor cortex and cerebellar network [13].

Despite numerous reports of cortical reorganization, the extent to which sensorimotor cortical representations are reorganized while attempting movements after SCI remains largely unknown, particularly with regard to changes in motor control‐related cortices and their functional cortical potential activity, which is a direct determinant of motor decoding performance. Accordingly, the main aim of this study is to investigate the changes in core cortical areas involved in motor control after SCI, as well as the changes in their potential activity. EEG signals were recorded during movement attempts or execution in SCI and healthy individuals. For the acquired data, we performed source localization analyses to examine the changes in core cortical regions related to motor control while attempting movements after SCI. In addition, the power spectral density (PSD) and event‐related spectral perturbation (ERSP) were also calculated to explore the characteristics of the changes in cortical potentials in motor regions. This study will reveal changes in core cortical areas involved in motor control after SCI, which is crucial for improving the accuracy of motor intention decoding in SCI patients and advancing the clinical application.

## 2 Materials and Methods

### 2.1 EEG Dataset Sources

In this study, two publicly available EEG datasets were used to examine the altered cortical activities and functional networks during upper limb movement in humans with SCI. Dataset 1 was from “Attempted arm and hand movements in persons with SCI” [14]. It included attempted hand movement EEG signals that were recorded from SCI patients and the sampling rate was 256 Hz. The dataset consisted of EEG data from 10 SCI patients between the ages of 20 and 78. With the exception of subject No. 4, all participants were male and all participants were right‐handed. Subject No. 7 was tested with the left hand, and the rest were right‐handed.

Dataset 2 was from “Upper limb movements can be decoded from the time‐domain of low‐frequency EEG” [15]. It included hand movements EEG signals that were recorded from healthy subjects and the sampling rate was 512 Hz. The data set consisted of EEG data from 15 healthy subjects aged 22 to 40 years. The average age of the subjects was 27 years old (standard deviation was 5 years), of whom 9 subjects were left‐handed females, except for subject No. 1 who was right‐handed. In our study, to ensure the consistency of variables, all subjects selected were right‐handed.

### 2.2 Data Collection Model

**FIGURE 1:**
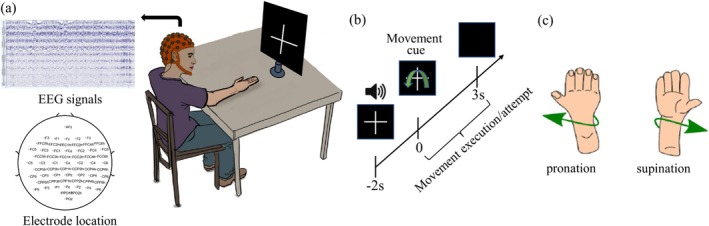
The experimental model for all the datasets. (a) Participants sat in front of a computer screen and performed hand movements. (b) The sequence of the model for all the datasets. (c) Illustration of the movements.

The two publicly available EEG datasets were all recorded from 61 channels using active electrodes. They use the same experimental model. Each of the subjects was wearing an EEG cap and sat in front of a computer screen with his/her arms on the table or supported by an exoskeleton with anti‐gravity support to avoid muscle fatigue. At the start of the trial, a fixation cross and a beep sound were presented. Each trial consists of 5 s. The first 2 s are the preparation time. The next 3 s require the subject to perform or attempt hand movements through a screen prompt. The experimental model is shown in Figure 1. Subjects focused on the cross displayed throughout the 5‐s experiment to avoid eye movements. A break of 2–3 s is provided between each trial. For Dataset 1, each subject performed 9 runs with 8 trials for each motion per run. For Dataset 2, each subject performed 10 runs with 6 trials for each motion per run.

### 2.3 EEG Data Processing

EEG data processing was performed using custom scripts written in MATLAB 2016a (The MathWorks) incorporating the EEGLAB 2020 toolbox [16].

#### 2.3.1 EEG Data Pre‐Processing

First, the EEG data were low‐pass filtered at a cut‐off frequency of 48 Hz (zero phase FIR, order 32) to remove high frequency noise. Then, the resulting data were down‐sampled to 256 Hz. Second, the data were high‐pass filtered using a filter with a cut‐off frequency of 3 Hz (zero phase FIR, order 1250) to remove the baseline drift. The electrodes indicated by a standard deviation greater than 1000 μV or a kurtosis of more than 5 standard deviations from the mean were defined as bad electrodes [17]. Bad electrodes were interpolated using spherical splines interpolation [18]. Spherical spline interpolation takes into account the volumetric conduction effect of scalp EEG and the weight contribution of electrodes at different distances, allowing for more effective compensation of bad electrodes. The data used for the spherical splines interpolation were those collected from eight electrodes surrounding the bad electrode in the scalp electrode distribution. Subsequently, the filtered data were re‐referenced to the common average reference.

#### 2.3.2 Localization of the Sources of EEG Data

First, independent component analysis (ICA) was used to separate the EEG data into brain activity, blink, and other artifacts [19]. The ICA algorithm employed in this study was Infomax ICA. The strength of Infomax ICA lay in its ability to separate statistically independent source signals in complex signal environments, allowing effective processing of EEG signals with non‐linear mixing characteristics [20]. The ICA decomposition was performed for each participant over all conditions (pronation and supination) using the “runica” function in EEGLAB [16]. The detailed parameter settings can be found in the “runica” function. Second, the independent component (IC) sources related to non‐brain activity were removed by inspecting each IC scalp projection and power spectra. For each participant and remaining IC, an equivalent current dipole located within a standardized three‐shell boundary element head model was estimated based on the Montreal Neurological Institute (MNI) standard brain [21, 22]. Then, for all participants, a template of electrode locations based on the MNI head model was used for source localization. The ICs were used for further analysis if their best‐fit equivalent current dipoles were located within the head and accounted for more than 85% of the variance seen at their scalp [23]. Subsequently, according to conditions (pronation and supination), the remaining ICs were divided into epochs of 5 s.

#### 2.3.3 Group Analyses of EEG Data

For the remaining ICs, the ERSP and PSD were calculated. Then, the ICs across the participants were clustered. For clustering of the ICs, feature vectors were created combining differences in dipole locations, scalp projection, and PSD. Those remaining ICs were clustered using the k‐means clustering algorithm. The number of clusters was set according to previous studies [24, 25]. ICs above three standard deviations from any of the resulting cluster centroids were identified as an outlier cluster and subsequently eliminated from analysis. Clusters containing less than half of the participants (≤ 5) were excluded from group analysis [26, 27]. Ultimately, five clusters were identified as brain sources responding to the forearm movement of SCI patients and eight clusters were identified as brain sources responding to the forearm movement of healthy subjects. For each cluster and condition, the average PSD was calculated. For each IC, a single IC ERSP was calculated. Then, the single IC cluster mean ERSP of each movement condition was obtained by averaging all IC ERSP. The bootstrapping technique within the EEGLAB toolbox was used to identify the significance of cluster ERSP and PSD (α = 0.05) between all conditions [16].

### 2.4 Statistics

In this study, the Shapiro–Wilk test was used to assess the normal distribution of cortical activity data. As the data did not exhibit normal distribution, the Wilcoxon rank‐sum test was used to evaluate the significant difference in cortical activities between SCI patients and healthy subjects for different movement states. The Wilcoxon rank‐sum test is a nonparametric statistical test for testing the difference between unpaired data. To account for multiple comparisons across 4 tests (4 frequency bands: theta band (3‐7 Hz), alpha band (7‐13 Hz), beta band (13‐30 Hz) and gamma bands (30‐45 Hz)), we applied the Bonferroni correction by adjusting the significance level to α = 0.05/4 = 0.0125.

## 3 Results

### 3.1 Independent Component Clusters

**FIGURE 2:**
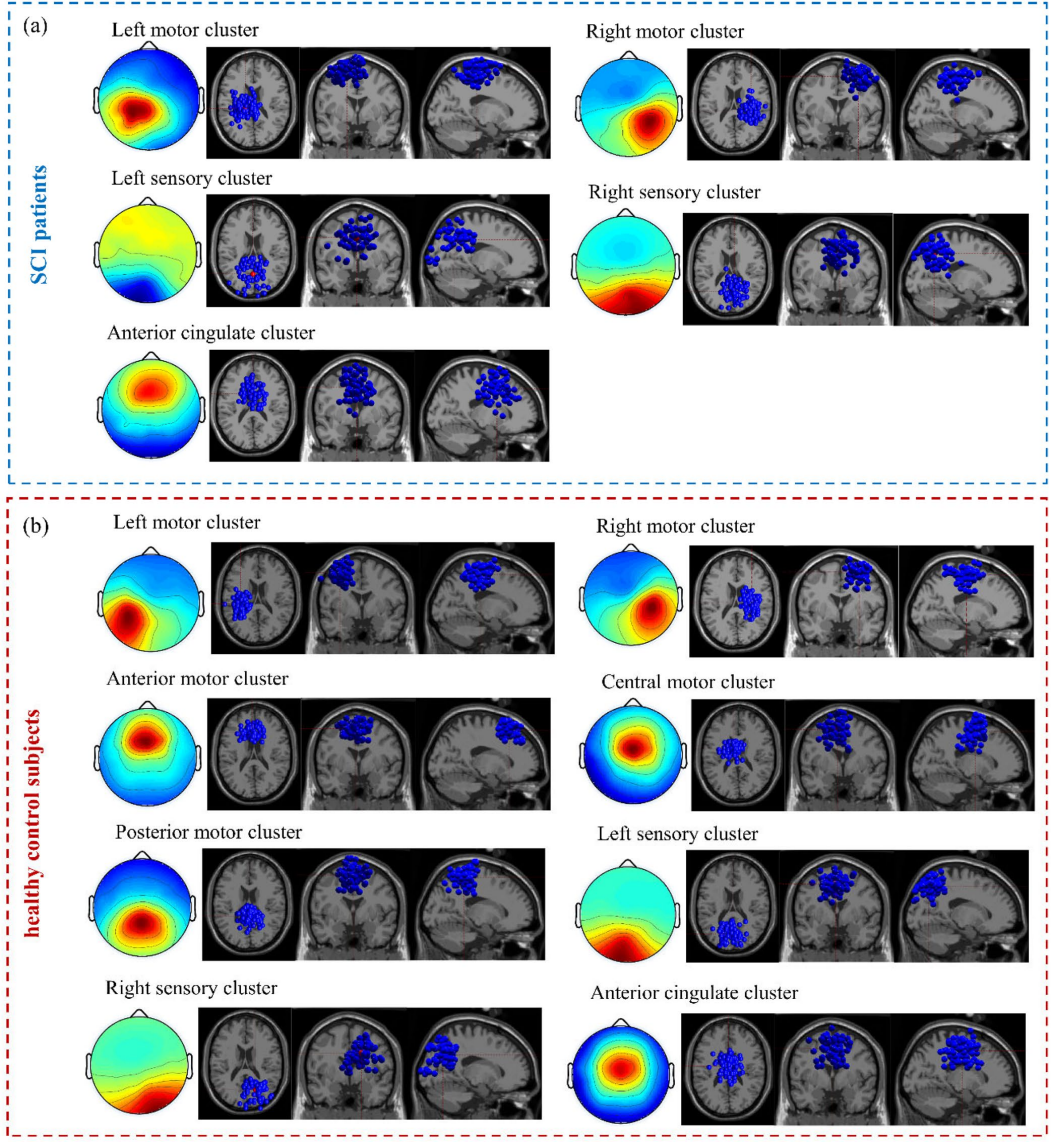
Dipole locations of IC clusters of SCI patients (a) and healthy control subjects (b). Each cluster is visualized in the Montreal Neurological Institute (MNI) brain volume in the top, coronal and sagittal views. Blue colored spheres indicate the dipole locations for single cortical sources for single participants, and red colored spheres indicate cluster centroids.

For SCI patients, five motor‐related clusters were located in the left motor, right motor, left sensory, right sensory, and anterior cingulate (Table 1, Figure 2a). While for healthy controls, eight motor‐related clusters were located in the left motor, right motor, anterior motor, central motor, posterior motor, left sensory, right sensory, and anterior cingulate (Table 1, Figure 2b).

**TABLE 1:** IC clusters of SCI patients.

	Cluster	No. of patients/ICs	Cluster centroid coordinates (*x*, *y*, *z*)	Brodmann area	Cortical location
SCI patients	Left motor	9/131 ICs	−16, −23, 64	BA4	Primary motor cortex
	Right motor	9/90 ICs	31, −25, 56	BA4	Primary motor cortex
	Left sensory	9/60 ICs	−1, −58, 35	BA7	Somatosensory association cortex
	Right sensory	9/100 ICs	8, −56, 43	BA7	Somatosensory association cortex
	Anterior cingulate	9/89 ICs	−1, 9, 41	BA32	Dorsal anterior cingulate cortex
Healthy control subjects	Left motor	9/124 ICs	−33, −24, 53	BA4	Primary motor cortex
	Right motor	9/122 ICs	29, −20, 57	BA4	Primary motor cortex
	Anterior motor	9/95 ICs	−4, 24, 52	BA6	Supplementary motor cortex
	Central motor	9/81 ICs	−8, −2, 52	BA6	Supplementary motor cortex
	Posterior motor	9/126 ICs	0, −35, 58	BA4	Primary motor cortex
	Left sensory	9/122 ICs	−2, −65, 49	BA7	Somatosensory association cortex
	Right sensory	9/97 ICs	19, −63, 33	BA7	Somatosensory association cortex
	Anterior cingulate	9/97 ICs	1, −8, 45	BA24	Ventral anterior cingulate cortex

### 3.2 Power Spectral Density

**FIGURE 3:**
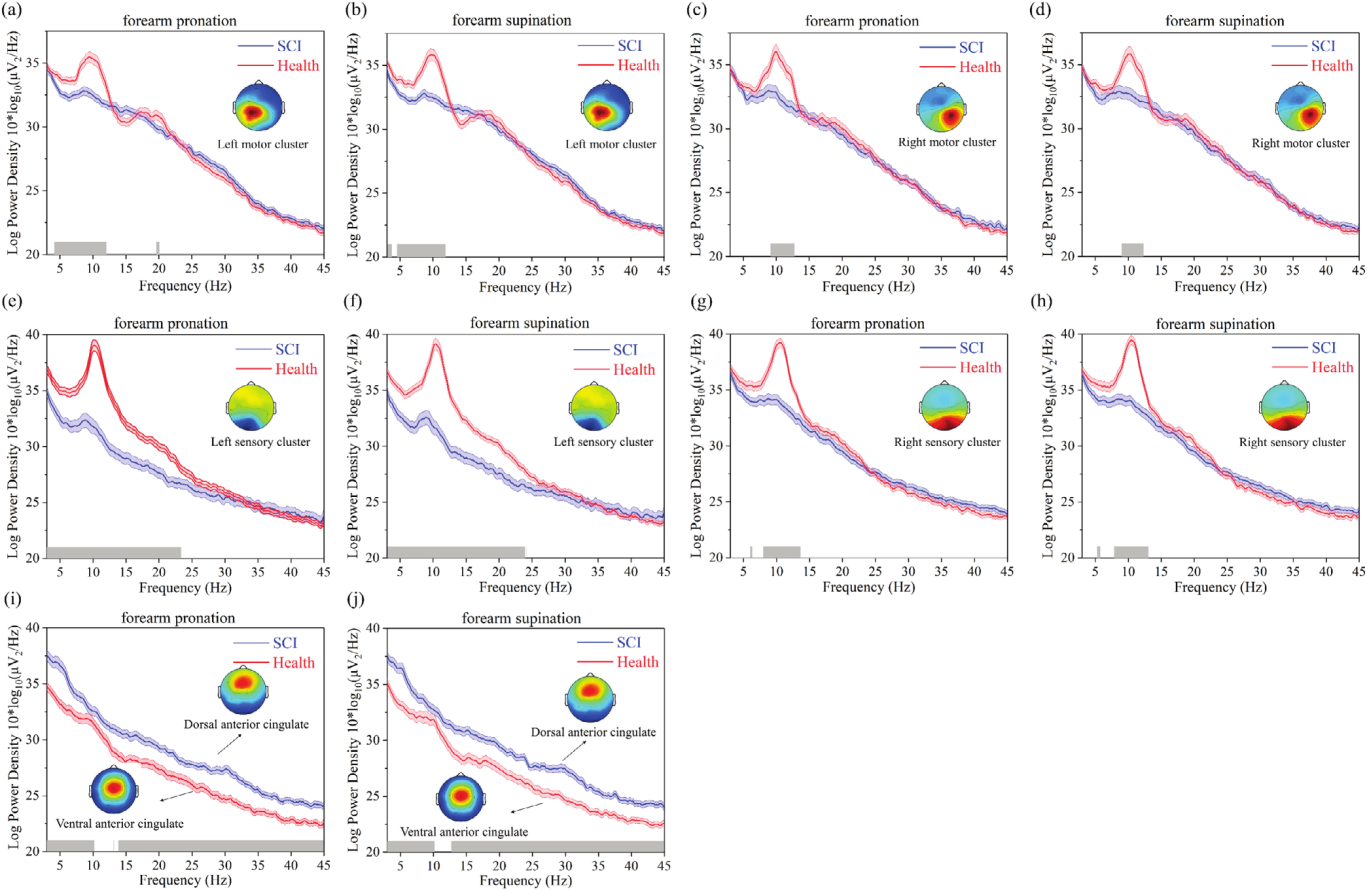
Power spectral density values of independent component clusters in the left motor, right motor, left sensory, right sensory and anterior cingulate of SCI patients and healthy control subjects, respectively. (a) Comparisons of the PSD values of the left motor cluster between SCI patients and healthy control subjects in the forearm pronation condition. (b) Comparisons of the PSD values of the left motor cluster between SCI patients and healthy control subjects in the forearm supination condition. (c) Comparisons of the PSD values of the right motor cluster between SCI patients and healthy control subjects in the forearm pronation condition. (d) Comparisons of the PSD values of the right motor cluster between SCI patients and healthy control subjects in the forearm supination condition. (e) Comparisons of the PSD values of the left sensory cluster between SCI patients and healthy control subjects in the forearm pronation condition. (f) Comparisons of the PSD values of the left sensory cluster between SCI patients and healthy control subjects in the forearm supination condition. (g) Comparisons of the PSD values of the right sensory cluster between SCI patients and healthy control subjects in the forearm pronation condition. (h) Comparisons of the PSD values of the right sensory cluster between SCI patients and healthy control subjects in the forearm supination condition. (i) Comparisons between the PSD values of the dorsal anterior cingulate cluster of SCI patients and the PSD values of the ventral anterior cingulate cluster of healthy control subjects in the forearm pronation condition. (j) Comparisons between the PSD values of the dorsal anterior cingulate cluster of SCI patients and the PSD values of the ventral anterior cingulate cluster of healthy control subjects in the forearm supination condition. The line represents the average PSD values in all ICs per cluster and the shading represents their standard error. The underbars (gray) indicate the frequency region of significant differences (Bonferroni corrected *p*‐value ≤ 0.0125).

The results revealed that the PSD in the theta band (3‐7 Hz) was obviously decreased in both the left and right motor clusters for SCI patients and healthy subjects (Figure 3a–d). However, compared with healthy subjects, the decrease in the PSD of SCI patients was greater in the left motor cluster (Bonferroni corrected *p*‐value = 0.00162) (Figure 3a,b), and the decrease in the PSD of the right motor cluster did not show significant differences (Bonferroni corrected *p*‐value = 0.453) (Figure 3c,d). In addition, compared to SCI patients, the PSD of healthy subjects in the alpha band (7‐13 Hz) was significantly increased in both the left (Bonferroni corrected *p*‐value = 0.000198) and right motor clusters (Bonferroni corrected *p*‐value = 0.000792) (Figure 3a–d). More unexpectedly, the trends of the PSD in the beta band (around 20 Hz) of the left and right motor clusters showed large differences. The PSD of the left motor cluster in the beta band was significantly decreased in SCI subjects (Bonferroni corrected *p*‐value = 0.00919) (Figure 3a).

Moreover, the results revealed that the PSD in the theta band was obviously decreased in both the left and right sensory cluster for SCI patients and healthy subjects (Figure 3e–h). However, compared to the right sensory cluster, the variability of the PSD in the left sensory cluster between SCI patients and healthy subjects was greater (Bonferroni corrected *p*‐value = 0.000145). Also, compared to SCI patients, the PSD of healthy subjects in the alpha band was significantly increased in the left sensory cluster (Bonferroni corrected *p*‐value = 0.000145). Moreover, the difference in PSD in the alpha band of the left sensory cluster between SCI patients and healthy subjects was greater (Figure 3e,f). The results also indicated that the PSD of the healthy subjects in the beta band (13‐23 Hz) was markedly larger than those of the SCI patients in the left sensory cluster (Bonferroni corrected *p*‐value = 0.000498) (Figure 3e,f).

**FIGURE 4:**
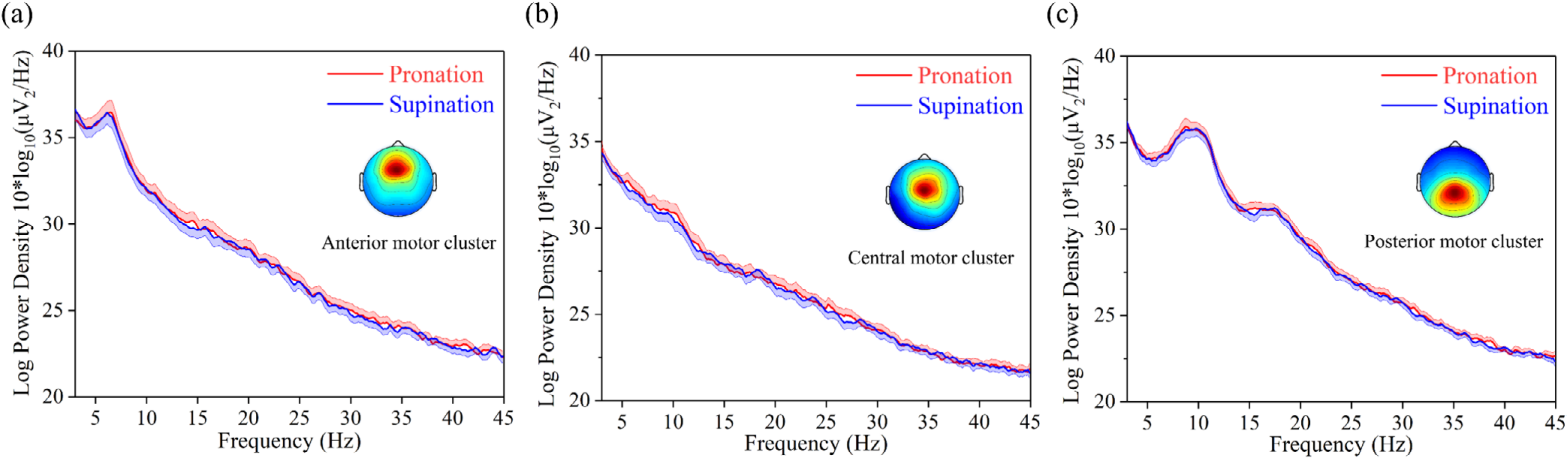
Power spectral density (PSD) values of independent component (IC) clusters in the anterior motor, central motor, and posterior motor of healthy control subjects, respectively. (a) The PSD values of healthy control subjects in the anterior motor cluster for forearm pronation and supination. (b) The PSD values of healthy control subjects in the central motor cluster for forearm pronation and supination. (c) The PSD values of healthy control subjects in the posterior motor cluster for forearm pronation and supination. The line represents the average PSD values in all ICs per cluster, and the shading represents their standard error.

The average PSD of the anterior cingulate cluster for SCI patients and healthy subjects is shown in Figure 3i,j. The difference is that in SCI patients, the activated area is the dorsal anterior cingulate cortex, and in healthy subjects, it is the ventral anterior cingulate cortex. Unlike the motor and sensory clusters, compared with PSD in healthy subjects, the PSD of the dorsal anterior cingulate cluster in the theta (Bonferroni corrected *p*‐value = 0.0000753), beta (Bonferroni corrected *p*‐value = 0.000152) and gamma bands (30‐45 Hz) (Bonferroni corrected *p*‐value = 0.0000418) is significantly larger in SCI patients (Figure 3i,j), except for the alpha band (Bonferroni corrected *p*‐value = 0.0321). This may indicate a compensatory effect on motor sensory function after SCI. Moreover, for healthy subjects, the PSD of the anterior motor cluster, central motor cluster, and posterior motor cluster also shows a different trend (Figure 4). The PSD of healthy subjects in the anterior motor cluster and central motor cluster shows an overall downward trend (Figure 4a,b). However, for the posterior motor cluster, the PSD in the theta band is decreased and in the alpha band is increased (Figure 4c).

### 3.3 Event‐Related Spectral Perturbation

**FIGURE 5:**
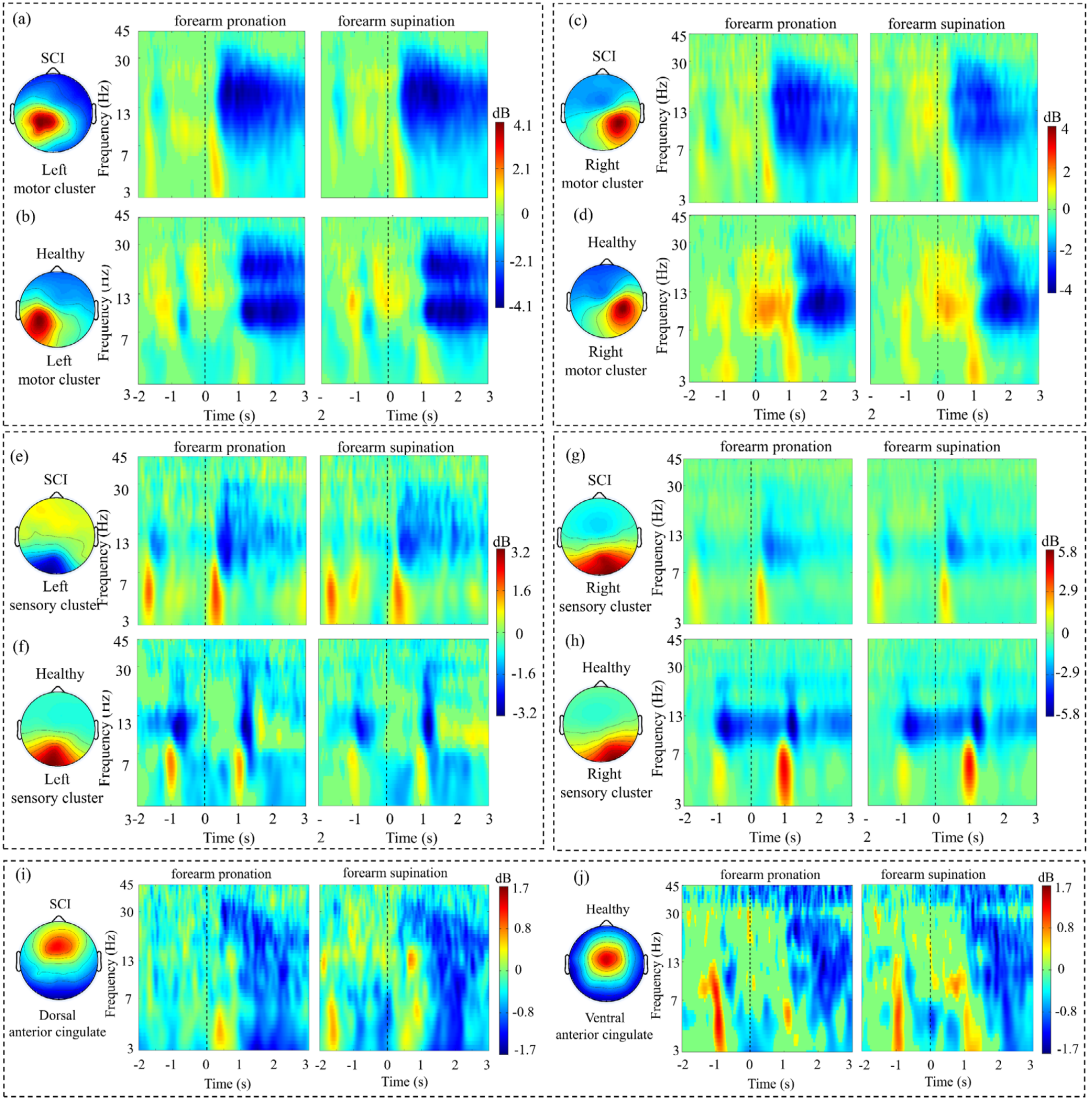
Event‐related spectral perturbation (ERSP) values of the left motor cluster, right motor cluster, left sensory cluster, right sensory cluster, and anterior cingulate cluster. (a) The average ERSP values of SCI patients in the left motor cluster. (b) The average ERSP values of healthy control subjects in the left motor cluster. (c) The average ERSP values of SCI patients in the right motor cluster. (d) The average ERSP values of healthy control subjects in the right motor cluster. (e) The average ERSP values of SCI patients in the left sensory cluster. (f) The average ERSP values of healthy control subjects in the left sensory cluster. (g) The average ERSP values of SCI patients in the right sensory cluster. (h) The average ERSP values of healthy control subjects in the right sensory cluster. (i) The average ERSP values of SCI patients in the dorsal anterior cingulate cluster. (j) The average ERSP values of healthy control subjects in the ventral anterior cingulate cluster. Warmer colors indicate a power increase, and the cooler colors indicate a power decrease. A timestamp of zero represents the beginning of the performing/attempting hand movement. The first 2 s represent the preparation phase; the next 3 s represent the phase of performing/attempting hand movement.

**FIGURE 6:**
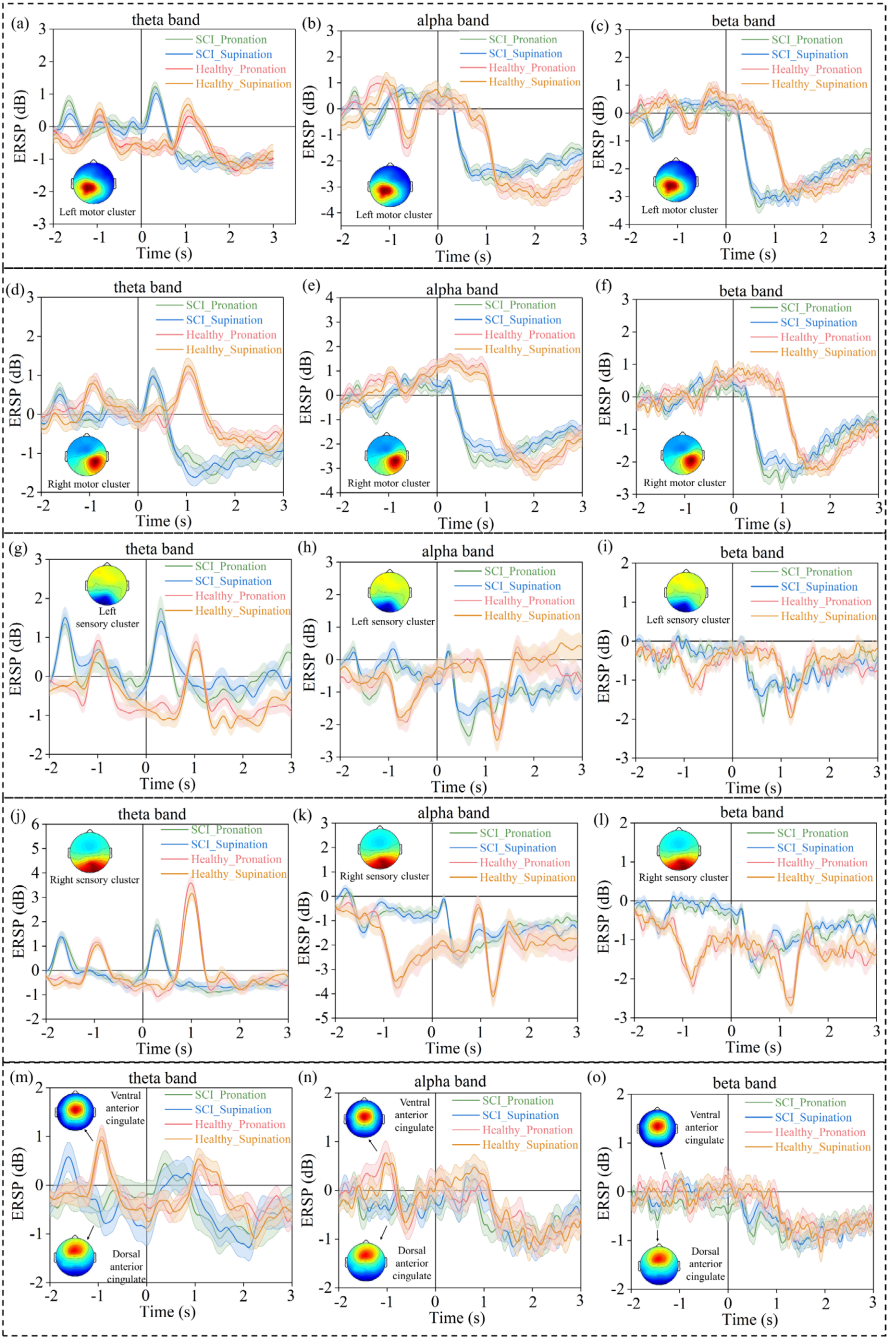
The theta band (3 to 7 Hz), alpha band (7 to 13 Hz) and beta band (13–30 Hz) spectral perturbation values of the left motor cluster (a–c), right motor cluster (d–f), left sensory cluster (g–i), right sensory cluster (j–l), and anterior cingulate cluster (m–o).

The results demonstrated that the modulation of the theta, alpha, and beta power depended on the movement phases in the left and right motor cortices, left and right sensory cortices, anterior cingulate cortex, as well as anterior motor cortex, central motor cortex, and posterior motor cortex clusters. In the left motor cluster, compared with healthy subjects, the theta band power appeared increased obviously at the beginning of the attempting movements phase for SCI patients (Figures 5a,b and 6a). Instead, the alpha band power obviously increased during the preparation phase for healthy subjects in the left motor cluster (Figures 5b and 6b). Additionally, the alpha and beta band power were all decreased for both SCI patients and healthy subjects in the left motor cluster during the phase of performing/attempting hand movement (Figures 5a,b and 6c). Unexpectedly, in the left motor cluster, the power modulation occurred earlier in SCI patients than in healthy subjects (Figure 6a–c).

Conversely, in the right motor cluster, the theta band power was increased during all phases for healthy subjects (Figures 5d and 6d). Also, as in the left motor cluster, the alpha band modulation showed an obvious increase at the movement beginning for healthy subjects (Figures 5d and 6e). Moreover, the alpha and beta band power were also obviously decreased for both SCI patients and healthy subjects in the left motor cluster (Figures 5c,d and 6e,f). Likewise, the power modulation occurred earlier in SCI patients than healthy subjects in the right motor cluster (Figure 6d–f).

In the left sensory cluster, compared to healthy subjects, the theta band power was increased for SCI patients at the beginning of the preparation phase and the attempting movement phase (Figures 5e and 6g). Additionally, the alpha band power was reduced during all the phases for both SCI patients and healthy subjects (Figures 5e,f and 6h). Instead, compared to SCI patients, the alpha band power was more obviously decreased during the preparation phase for healthy subjects (Figures 5f and 6h). Moreover, the beta band power was also decreased during the phase of performing/attempting movement for both SCI patients and healthy subjects (Figures 5e,f and 6i).

The ventral anterior cingulate cluster showed obvious theta and alpha band power increases for healthy subjects during all phases (Figures 5j and 6m,n). In contrast, the dorsal anterior cingulate cluster showed a theta band power decrease for SCI patients (Figures 5i and 6m). During the phase of performing/attempting movement, the above two clusters all showed alpha band power and beta band power decreases (Figures 5i,j and 6n,o).

**FIGURE 7:**
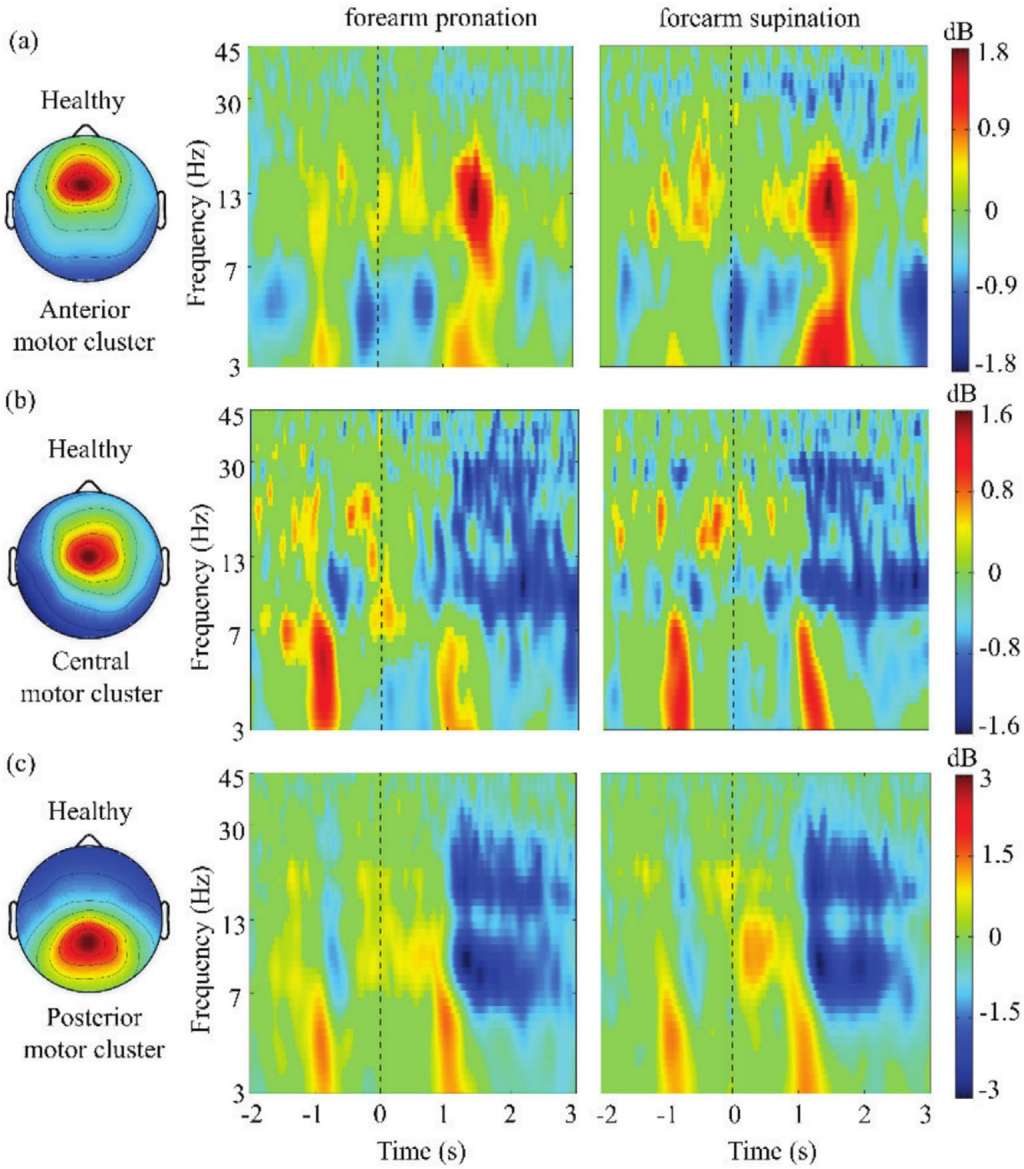
Event‐related spectral perturbation (ERSP) values of the anterior motor cluster (a). Event‐related spectral perturbation (ERSP) values of the central motor cluster (b). Event‐related spectral perturbation (ERSP) values of the posterior motor cluster (c).

The results additionally demonstrated that other clusters of healthy subjects also showed theta, alpha, and beta power modulation depending on the movement phases (Figure 7a–c). In the anterior motor cluster, the theta and alpha band power were increased (Figure 7a). The central motor cluster also showed increased theta band power (Figure 7b). In the posterior motor cluster, the theta band power was increased during all phases (Figure 7c). In addition, the alpha and beta band power were decreased during the performing movement phase.

## 4 Discussion

### 4.1 Reduced Motor‐Related IC Clusters of Cortical Activation in SCI Patients

Compared to healthy subjects, motor‐related IC clusters were obviously decreased while attempting upper limb movement in SCI patients (Figure 2). It was obvious that the activated areas of the motor cortex were reduced in SCI patients, with only the primary motor cortex being activated, while in healthy subjects, in addition to the primary motor cortex, the supplementary motor cortex was also activated (Table 1). Our results also showed that primary motor cortex areas were reduced during voluntary forearm movements after SCI (Table 1). A previous study reported reduced subcortical white matter volume and cortical gray matter volume in the corticospinal tract and cortical thinning in the primary motor cortex in patients after SCI [28]. This may be due to spinal cord atrophy after SCI, resulting in reduced dendritic spine density [29] or weakened angiogenesis, leading to reduced cortical connectivity. Another study showed that the motor map area in a hand muscle decreased with voluntary contraction in SCI patients, possibly due to sensory deficits [30]. A study in monkeys also showed that the ipsilesional motor cortex showed substantial reduction of the hand representation after cervical hemi‐section [31]. The paradoxical reduction of hand representation in the ipsilesional hemisphere was secondary to the changes taking place in the contralesional hemisphere, possibly corresponding to postural adjustments or re‐establishment of the balance between the two hemispheres [31].

Additionally, the results also showed a reduction in the activation of the supplementary motor cortex after SCI (Table 1). This was consistent with previous findings that SCI patients had obviously decreased gray matter volume in the bilateral supplementary motor cortex compared to healthy control subjects [32]. This may be related to the specific function of the motor cortex areas, where neurons in the primary motor cortex are thought to control muscles directly via spinal motor neurons, and the supplementary motor areas, which play a role in planning and coordinating movement. After SCI, the basic motor control functions of the primary motor cortex are preserved, but motor planning and coordination are lost.

### 4.2 Sensorimotor Cortex Activity Weakened During Upper Limb Movement in SCI Patients

The sensorimotor cortex showed that the average PSD of the theta, alpha, and beta bands was obviously decreased during upper limb movement in SCI (Figure 3). This was consistent with a previous functional magnetic resonance imaging‐based study of sensorimotor cortex activation in paralyzed patients after SCI showing that activation of the primary motor cortex and associated cortical sensorimotor areas decreased progressively with increasing duration of paralysis compared to healthy subjects [33]. In detail, the PSD in the theta band was obviously decreased in both the left motor cluster and right motor cluster for SCI patients compared to healthy subjects, while for the PSD in the alpha band, the increases were obviously lower in SCI patients than in healthy subjects (Figure 3a–d). The results also demonstrated that the PSD of the theta and alpha bands in the left and right sensory cortices showed similar trends (Figure 3e–h). Unexpectedly, the PSD in the beta band of the left motor cluster showed large differences between SCI patients and healthy subjects. Moreover, the PSD of the beta band in the left sensory cluster was markedly lower in SCI patients than those in healthy subjects. Reduced activity in the sensorimotor cortex may be a potential biomarker for loss of processing and sensorimotor integration of motor planning/execution after SCI [34], as a direct response to motor performance at the cortical level.

Furthermore, the anterior cingulate cortex was activated during attempted limb movements in SCI patients in the same way as in healthy subjects (Figure 2). The difference was that in SCI patients the activated area was the dorsal anterior cingulate cortex, while in healthy subjects it was the ventral anterior cingulate cortex (Table 1). The ventral anterior cingulate cortex was activated during motor execution in healthy subjects [35], and the change in the location of activation in the anterior cingulate cortex in the present study may be due to functional reorganization of the cortex after SCI. Unlike the sensorimotor cortex, the PSD of the dorsal anterior cingulate cluster in the theta, alpha, beta, and gamma bands was obviously larger in the SCI patients than in healthy subjects (Figure 3i,j). It is well known that the motor‐related functions of the anterior cingulate cortex include attentional processing, movement monitoring, and error detection and correction [36]. Thus, the increased PSD may be due to the requirement of a more focused internal attention process by SCI patients.

Notably, compared to SCI patients, the supplementary motor cortex and additional areas of the primary motor cortex were also activated in healthy subjects (Table 1). The PSD of healthy subjects in the anterior motor cluster and central motor cluster showed an overall downward trend (Figure 4a,b). However, in the posterior motor cluster, the PSD in the theta band was decreased and in the alpha band was increased (Figure 4c). This finding revealed the details of the supplementary motor cortex in motor control.

### 4.3 Obvious Changes in Cortical Power Modulation After SCI

Compared to healthy subjects, cortical power modulation was obviously altered in SCI patients while attempting upper limb movement, including theta, alpha, and beta power modulation (Figure 5). This may be an adaptive adjustment following functional reorganization of the sensorimotor cortex of the brain [37]. Obvious increases in theta band power in the left motor cluster and left sensory cluster occurred at the beginning of the preparation and attempting movement phase in SCI patients (Figures 5a,e and 6a,g). Conversely, in the right motor cluster, right sensory cluster, and anterior cingulate cluster, more obvious increases in theta band power occurred in healthy subjects than in SCI patients (Figures 5d,h,j and 6d,j,m). These results revealed detailed changes in theta band power modulation in the sensorimotor‐related cortex after SCI, reflecting the result of the functional reorganization of the cortex. Previous studies have found increased intracranial theta band power during the planning phase of a capture task [38], the planning and execution phases of a choice response task [39], and during motor imagery for sensorimotor planning [40]. Local field potential studies in monkeys have also revealed increased theta‐band activity in the motor cortex during movement planning and execution, with activity correlating with the intended direction of the movement, suggesting that theta activity can be used to optimize performance by encoding precise task details [41]. This is consistent with our findings that increases in the theta band power of the sensorimotor cortex occurred during upper limb movement preparation and execution. In addition, theta oscillations were more prevalent during motor initiation and execution compared to rest periods, which may represent a broadly applicable rhythmic mechanism for sensorimotor integration in the human brain [42]. Therefore, a reasonable hypothesis was that the abnormal power modulation in the theta band of the sensorimotor cortex after SCI may indicate abnormal sensory operant integration function as well as impaired fine motor control.

We also found that the alpha band power was increased during the preparation and beginning phase for healthy subjects in the right motor cluster (Figures 5d and 6e). We interpreted the relative increase in ipsilateral hemispheric alpha‐band power as a suppression of neuronal populations that interfere with the demands of the task at hand [43]. This interpretation is consistent with the notion that alpha‐band oscillations implement inhibitory gating [44]. Within this framework, stronger oscillations in the alpha band are a mechanism for inhibiting neural processing in cortical areas that are not relevant to the task. Moreover, all the alpha band power decreased for both SCI patients and healthy subjects in all sensorimotor clusters and the anterior cingulate cluster during the performing/attempting hand movement phase (Figure 5). Decreases in the alpha band power were usually driven by processes unrelated to sensorimotor processes, such as changes in power driven by changes in visual input or arousal [43]. This was consistent with our findings that the decrease in the alpha band power during locomotion may be driven by visual cues. Notably, there was a decrease in alpha band power in the left and right sensory clusters during the preparation phase (Figures 5f,h and 6h,k). This finding supported previous findings that a decrease in the alpha band power occurred in the primary sensorimotor region prior to motor initiation [38], possibly in response to the process of exercise planning and preparation. Thus, a possible explanation for the altered power modulation in the alpha band may be that motor initiation becomes more difficult due to impaired motor function after SCI, but maintenance of movement after initiation is largely unaffected.

Unexpectedly, all sensorimotor clusters and the anterior cingulate cluster showed beta band power decreases both in SCI patients and healthy subjects during the performing/attempting hand movement phase (Figure 5). This is consistent with previous studies showing a decrease in the beta band power in bilateral sensorimotor and parietal areas during motor execution [39]. It has been theorized that beta band activity serves to promote the motor status quo, or that the motor cortex integrates the beta band cortical activity from parietal areas in the current situation for accurate motor performance [45]. In other words, the broadband oscillatory coordination in the beta band facilitates precise movement of the upper limb. Thus, the reduction in beta band neural oscillations may mediate the activity of neuronal populations calculated with motor parameters, thereby facilitating the maintenance of movement [46]. Notably, our study revealed that cortical activity in the beta band did not show obvious differences between SCI patients and healthy control subjects. From another perspective, it was shown that motor execution was not affected once initiated after SCI.

Another important finding of this study was the existence of an obvious difference in the latency of the largest power increase or attenuation between SCI patients and healthy subjects. To the best of our knowledge, this finding has not been reported in previous studies. In detail, the largest theta band power increase, the largest alpha band power attenuation, and the largest beta band power attenuation all occurred earlier in SCI patients than in healthy control subjects in all sensorimotor clusters (Figure 6). The changes in latency indicated altered somatosensory processing after SCI. One possible explanation is that movement attempts after SCI required more attention, inducing a pioneering change in power modulation in the sensorimotor cortex. It should be noted that this is only a hypothesis based on the results, and a deeper understanding of the mechanisms requires further studies.

## 5 Conclusion

This study investigated the changes in core cortical areas involved in motor control after SCI, as well as the changes in their cortical potential activity. Our results showed that motor‐related IC clusters obviously decreased while attempting upper limb movement after SCI, including the reduction in activated areas of the primary motor cortex and the absence of the supplementary motor cortex. Additionally, the activities in the sensorimotor cortex were obviously attenuated after SCI. This study also found that theta, alpha, and beta power modulation was obviously altered in SCI patients while attempting upper limb movement. Another remarkable finding was the existence of an obvious difference in the latency of the largest power increase or attenuation between SCI patients and healthy control subjects.

Although our study demonstrates some detail of the changes in motor sensorimotor cortical activities after spinal cord injury, there are some limitations. First, we only explored the altered sensorimotor cortical activities during forearm movement after spinal cord injury, not examining changes in functional connectivity between the different sensorimotor cortices. As the most complex and efficient system in nature, the brain requires not only a functional division of labor between different brain regions to carry out its functions, but also synergistic cooperation between different regions to form a complex functional network to fulfill its role. Therefore, an important component of future research is to investigate the obvious differences in brain connectivity between distributed brain regions during forearm movement in humans after spinal cord injury. Second, it remains unknown whether neuromodulation, such as ultrasound, can be used to modify the pattern of activation in the sensorimotor cortex to improve the accuracy of motor intention decoding.

Overall, our findings revealed changes in core cortical areas involved in motor control after cortical reorganization in SCI patients, which can be used to assess impaired motor function after spinal cord injury from the neurophysiological perspective. In addition, these results can also be used to assess the therapeutic efficacy of motor rehabilitation using spinal nerve electrical stimulation to improve treatment strategies. For patients with paralysis due to spinal cord injury, these results can also be used to guide deep learning decoding algorithms to improve BCI performance for more precise control of external devices.

## Author Contributions

Conceptualization: B.L.; Methodology: B.L., Y.L., and R.T.; Software: G.L.; Writing: B.L.; Review and editing: G.L. and H.L.; Project administration: F.Y.; Funding acquisition: B.L. All authors have read and agreed to the published version of the manuscript.

## Conflicts of Interest

The authors declare no conflicts of interest.

## Data Availability

The data and materials supporting the results in this article are available from the corresponding author on reasonable request.
